# Methodological Issues in Diagnostic Studies

**DOI:** 10.4103/1319-3767.77253

**Published:** 2011

**Authors:** Ahmed A. Sarkhy

**Affiliations:** Department of Pediatric, Gastroenterology Unit, King Khalid University Hospital, King Saud University, Saudi Arabia E-mail: asarkhy@hotmail.com

Sir,

I have read with great interest the article by Dehdashti *et al* utilizing the sonographic measurement of abdominal esophageal length as a tool towards the diagnosis of gastroesophageal reflux disease (GERD) in infants and neonates.[1] The study found that neonates and infants with reflux had a significantly shorter abdominal esophagus than subjects without reflux.However, this study has several critical methodological flaws that I would like to summarize in the following points:

1. In conducting any study on the accuracy of a diagnostic test, the new test should be compared with the prevailing ‘gold standard’. In this study, barium meal was used as the comparative test, although it is not the gold standard test for GERD diagnosis. Barium meal has poor sensitivity and specificity in detecting GERD, with its sensitivity reported to range from 29 - 86% and specificity from 21 - 83%, when compared with esophageal pH monitoring, which in turn is considered as the current gold standard for GERD diagnosis.[2] The main utility for the barium meal in GERD is to rule out anatomical abnormalities, such as esophageal stricture, achalasia, tracheoesophageal fistula or malrotation as possible causes for recurrent vomiting. Recent recommendations of the North American Society for Pediatric Gastroenterology, Hepatology and Nutrition do not support using barium meal to diagnose GERD.[2]
2. As mentioned in the study, sonographic detection of GERD mainly depends on the detection of returning of gastric fluid to the esophagus. This finding is not necessarily pathological. We know from previous studies that regurgitation occurs physiologically in up to 50% of the infants younger than 3 months and reduce with their growth, till spontaneously resolving by the age of 12–18 months.[3] In infants presenting with regurgitation or vomiting, using this sonographic finding would not really differentiate between physiological gastroesophageal reflux and pathological GERD.
3. Monitoring the esophageal pH is the current gold standard for diagnosing GERD, although, it does have its own limitations. One of these limitations is that it is designed to detect only acidic reflux (ie, pH less than 4). In fact, even comparing sonographic findings to pH monitoring is neither sufficient nor appropriate since ultrasound measures the number of refluxes, whereas pH study measures the amount of acidic reflux and the duration of its exposure with esophageal mucosa. The new modality of combined pH–multichannel intraluminal impedance study is a promising diagnostic modality that may replace the current gold standard in the near future, once the normal values in different age groups of children are validated.[4,5]
4. The infants enrolled in the study as GERD cases did not have a uniform diagnostic modality for their assumed GERD. Some of those infants were diagnosed clinically (with the associated GERD complication), while others had endoscopically or histologically proven diagnosis. The presence or absence of GERD should be uniformly documented in each infant enrolled in the study (either in the case or the control groups) using the gold standard test; otherwise misclassification between cases and control would occur eventually.
5. The authors excluded patients who were exposed to acid suppressive therapy within 24 h prior to the sonographic study and included those who were exposed within 48 or 72 h prior to the test. It is not expected for acid suppressive medications, such as proton pump inhibitors to lose their effect within 24 h, and therefore at least a washout period of 3 – 5 days should have been mandated before enrollment in order to minimize the possibility of having false-negative results.
6. Classifying GERD severity to mild, moderate, or severe according to the number of refluxes over 10 min is not appropriate. Severity of reflux is typically identified according to the reflux index, the contact time of acidic reflux with esophageal mucosa, and the extent of the refluxate along the esophagus; these can be objectively by the double-sensor pH probe study. As mentioned above, measuring the number of refluxes by sonography and comparing its results with a nonstandard test (barium meal) is not appropriate and subsequently the correlation between esophageal length and GERD diagnosis is not valid, and therefore the final conclusion of the authors that sonography could be considered as a single and adequate test for GERD is eventually invalid.
7. Finally, for any diagnostic study, it is recommended to report the findings in terms of sensitivity, specificity, positive predictive value, and negative predictive value and likelihood ratios for positive and negative tests. These are measures of diagnostic accuracy for any new diagnostic modality being evaluated. In addition, to ensure validity of the new test, inter- and intraobserver variability needs to be documented. If such a test gives different results by different people or in different occasions, it can hardly be useful.
